# Small-Holdings and Hospitals

**Published:** 1921-01-22

**Authors:** 


### Small-holdings and Hospitals.

ALTHOUGH the cultivation of land in this country is now fairly general, there are probably few who realise what a large part the small-holding can play among the sick in hospital at Christmas. It is, perhaps, not too much to assert that one of the chief events is the Christmas dinner, with its turkeys and fowls, with their etcetera of hams and sausages; those who spend their time in poultry-keeping and pig-rearing would be inspired were they to see patients indulging in the seasonable fare—men, women, and children, who in the ordinary way would be on a fish or special diet, are eating heartily and seem to be none the worse for the experience, while, of course, the plum pudding, with its numerous eggs, is enjoyed by all; and small wonder that enjoyment is there to be found when one thinks of the wonderful wards decorated and made veritable fairy glens with the flowers that come from greenhouses and warm borders and with the holly and evergreens cut from carefully tended bushes, to say nothing of the multitude of other things—apples and pears from the orchards—jam made from the plums and the currents—the honey from the hives, all of which find their way to every hospital at this time of the year. Thus might many be glad and interested to know the part their little piece of land plays in bringing happiness and cheer to many sick and suffering at the festive season of Christmas.

